# A Systematic Review of Evidence-Based Health Benefits of *Oroxylum indicum* and Its Functional Food Potential

**DOI:** 10.3390/foods14203465

**Published:** 2025-10-10

**Authors:** Hai Linh Nguyen, Amporn Sae-Eaw, Dinh Quyen Tran, Witoon Prinyawiwatkul, Yaowared Chulikhit

**Affiliations:** 1 Department of Food Technology, Faculty of Technology, Khon Kaen University, Khon Kaen 40002, Thailand; hailinh.n@kkumail.com (H.L.N.); sampor@kku.ac.th (A.S.-E.); 2 Department of Chemistry, Trent School of the Environment, Trent University, Peterborough, ON K9L 0G2, Canada; quyentran@trentu.ca; 3 School of Nutrition and Food Sciences, Agricultural Center, Louisiana State University, Baton Rouge, LA 70803, USA; wprinya@lsu.edu; 4 Division of Pharmaceutical Chemistry, Faculty of Pharmaceutical Sciences, Khon Kaen University, Khon Kaen 40002, Thailand

**Keywords:** *Oroxylum indicum* (L.) Benth. ex-Kurz, flavonoids, baicalein, bibliometric analysis, ADO framework, ethnomedicine, pharmacology, bitterness, functional food

## Abstract

*Oroxylum indicum* is a traditional food in Asia rich in flavonoids and health-related effects. However, research into the use of *O. indicum* as a functional food ingredient is overlooked. This review synthesized literature from 2010 to 2025 using the PRISMA framework, bibliometric mapping, and the Antecedents–Decisions–Outcomes (ADO) model. In total, 185 articles were included, covering in vitro, in vivo, in silico, and limited human investigations. Bibliometric analysis shows a growing global interest, with recent focuses on molecular docking, cancer, and conservation. Phytochemical investigations consistently report key flavonoids, including baicalein, baicalin, oroxylin A, chrysin, oroxin A, and oroxin B. Studies have linked these compounds to different health benefits, including antioxidants, cardiovascular, and neurological protection. Application of the ADO framework organized research into motives, methods, and outcomes, and highlighted scientifically validated ethnomedicinal uses, such as diabetes and liver protection. Meanwhile, gaps, including obesity-related studies, long-term safety, and clinical trials, remain. More importantly, although young fruits are locally consumed as vegetables or beverages, translation into functional foods is limited by bitterness, lack of standardized preparations, and insufficient dose–response data at dietary intakes. This review discusses the bioactive potential of *O. indicum* and outlines research priorities for its development in functional food.

## 1. Introduction

*Oroxylum indicum* (L.) Benth. ex-Kurz (*O. indicum*) belongs to the family Bignoniaceae and is a culturally important Asian plant. The tree is traditionally consumed as both food and medicine. Young fruits and shoots are eaten as vegetables in Thailand and neighboring countries, whereas the seeds are sometimes used in herbal drinks [1]. Beyond its local dietary use, stems and roots are decocted to treat asthma, diarrhea, bronchitis, and some respiratory problems [2].

Although many ethnomedicinal uses have been reported, some claims are still scientifically unconfirmed. Traditional applications for conditions such as asthma, diarrhea, jaundice, and wound healing are frequently documented in local knowledge systems [3,4,5,6], but mapping between scientific pharmacological effects and conventional uses is lacking. This raises an important question of whether such uses reflect genuine bioactivity or are anecdotal traditions. The identification of validated versus unsubstantiated effects is vital prior to the deployment of *O. indicum* as a source of bioactive compounds for functional foods.

In recent decades, *O. indicum* has gained attention for its rich flavonoids associated with health benefits. Nagasaka et al. [7] determined p53 activation and anticancer effects of chrysin isolated from the bark. Meanwhile, Zhang et al. [8] demonstrated antidiabetic activities of baicalein. These findings explain why the plant is valued in both traditional medicine and modern research on functional foods. However, its use in food products is still constrained by bitterness, a lack of standardized formulations, limited safety, and bioavailability at nutritional intake levels.

In the pharmaceutical context, many studies on *O. indicum* have examined pharmacological effects but rarely cover the full range of methods. Most are limited to in vitro assays, which show cellular activity [9,10,11,12]. Some perform in silico models, which predict compound–target interactions [13,14,15,16]. While valuable, these approaches cannot completely establish efficacy. More biologically relevant evidence comes from in vivo or ex vivo studies, which reflect metabolism, immunity, and absorption [8,17,18,19]. However, these are scarce. This review categorizes the available studies based on their methodological approaches to show which health effects have been biologically validated. It also reveals gaps, especially in linking in vitro/in silico findings with in vivo and clinical validation.

There are several existing reviews on *O. indicum*, yet many are outdated or too narrow. Some older narrative reviews looked at its traditional and medical uses, while more recent systematic reviews focused only on a specific compound or a medicinal effect [20,21,22]. No comprehensive review has integrated research trends, mapped traditional uses with scientific health benefits, and evaluated the plant’s bioactive chemistry for functional food potential. This paper presents a systematic synthesis of studies on *O. indicum* from 2010 to 2025. The PRISMA framework and bibliometric mapping were first used to identify trends in publication output, geographic locations, and thematic focus. To move beyond descriptive patterns, the Antecedents–Decisions–Outcomes (ADO) framework was applied to interpret the literature [23]. It helps clarify why studies were conducted (antecedents), how they were designed (decisions), and what results were obtained (outcomes). This integration separates ethnomedicinal and pharmaceutical research, verified from unsubstantiated claims, and highlights gaps, including insufficient safety data, dose–response studies, and clinical validation. The review also identifies the flavonoids most consistently linked to health benefits and discusses barriers, such as bitterness and standardized formulations, that restrict translation into functional food products.

The review answers the three main research questions. (1) What are the research trends and thematic developments of studies on *O. indicum* between 2010 and 2025? (2) What are scientifically validated and unsubstantiated ethnomedicinal uses? (3) What barriers and opportunities shape the translation of *O. indicum* into functional food applications, particularly with respect to safety, standardization, and sensory properties?

## 2. Methodology

**Figure 1:**
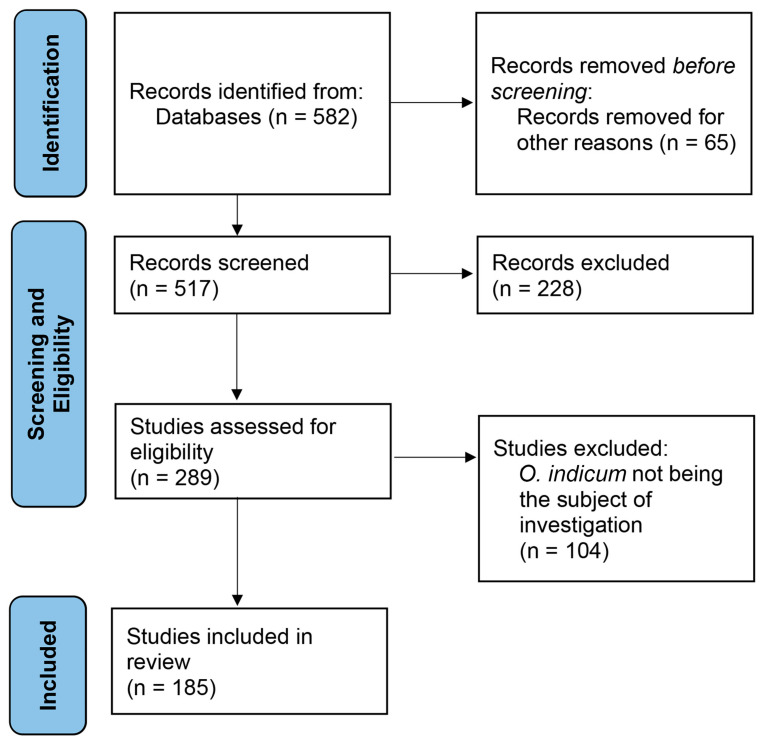
PRISMA 2020 flowchart for the selection of studies on *O. indicum*.

The PRISMA framework (2020 guidelines) was used to select relevant papers with research identification, screening, eligibility, and inclusion (Figure 1).

### 2.1. Identification

The Scopus database was searched on 5 April 2025 for studies on *O. indicum* between 2010 and April 2025. The search included both the scientific name and common names as keywords in titles, abstracts, and author keywords: “*Oroxylum indicum*” OR “midnight horror” OR “Indian trumpet flower” OR “broken bones plant” OR “Tree of Damocles” OR “Indian calosanthes” OR “Indian caper” OR “Indian trumpet” [20,24]. A total of 582 records were initially retrieved. Inclusion criteria were peer-reviewed journal articles, reviews, conference papers, and data papers published in English. Exclusion criteria eliminated non-peer-reviewed sources (e.g., book chapters, reports, editorials, working papers) and records from subject areas unrelated to life, health, or agricultural sciences (e.g., mathematics, computer science, economics). After applying these criteria, 517 articles remained, with 65 records removed. Filtering was performed using Scopus’ built-in tools.

### 2.2. Eligibility and Screening

Articles were assessed using Scimago Journal Rank (SJR) quartiles, and only quartile (Q1 and Q2) studies were considered eligible. After this eligibility, 289 studies remained and 228 were excluded. The shortlisted papers were then screened manually for relevance to *O. indicum*. Although the initial keyword search included terms such as “Indian trumpet” and “midnight horror,” some results referred to unrelated species, which were removed. Data from each included study was extracted separately by two reviewers using a standardized data extraction sheet. Discrepancies were resolved through discussion. Following this process, 104 additional articles were excluded, resulting in 185 research articles included for analysis.

### 2.3. Inclusion

The 185 research articles were analyzed using bibliometric methods and interpreted through the ADO framework. For ADO analysis, outcomes were extracted from each study individually, and studies were grouped into predefined categories according to the objectives. Each article could be assigned to one or more categories. Bibliometric analyses were conducted in RStudio (2024.12.0-467) running R version 4.4.2 with the bibliometrix package (version 5.1.1) [25], and a keyword co-occurrence network map was generated with VOSviewer 1.6.20 [26].

### 2.4. Limitations

This review used only one database (Scopus), but it is widely accepted in bibliometric and systematic reviews for its broad coverage and consistent metadata. Focusing on Q1 and Q2 journals may leave out some relevant studies, yet it helped highlight reliable research trends from higher-quality sources. In addition, the connections between traditional uses and pharmacological effects were based on plausible biological links, not direct scientific proof. In some cases, the absence of traditional context made interpretation limited. In the keyword analysis, six articles (3.24% of the total) did not list author keywords, but this small proportion is unlikely to affect the overall trends.

## 3. Results

### 3.1. Bibliometric Landscape

#### 3.1.1. Publication Trends

**Figure 2:**
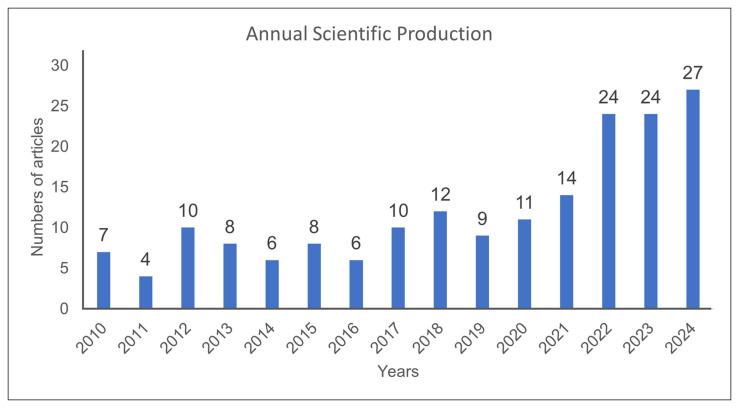
Growth of annual publications related to *O. indicum*.

Figure 2 shows the annual scientific output on *O. indicum* from 2010 to 2024. Publication numbers remained relatively low, with fewer than 15 papers per year until 2021. A sharp rise occurred in 2022, when annual output reached 24 articles, followed by 24 in 2023 and a peak of 27 in 2024. This upward trajectory indicates the growing research attention to *O. indicum* in recent years. While the figure captures the scale of publication activity, the specific research directions and thematic developments are examined in the subsequent bibliometric and ADO analyses.

#### 3.1.2. Geographical Distribution

**Figure 3:**
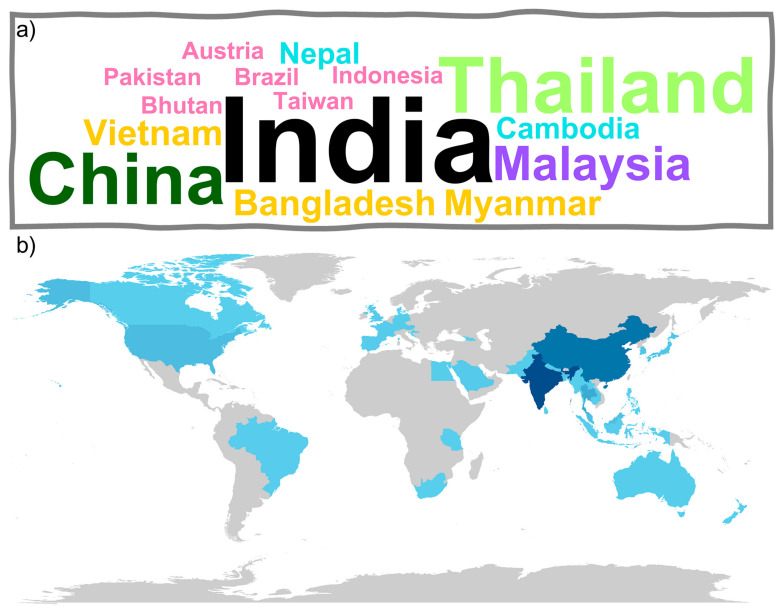
(**a**) Countries where *O. indicum* samples were collected in the selected papers and (**b**) the distribution of author affiliations by country across all selected publications related to *O. indicum*. In (**a**), countries with similar colors indicate comparable numbers of sample collections, while in (**b**), darker colors are countries with more author affiliations, and lighter colors indicate fewer records.

Figure 3a shows the countries where *O. indicum* samples were collected for investigation. Most collections were reported in India, Thailand, and China, which reflects the species’ native distribution and its cultural importance in South and Southeast Asia. Notably, Cardoso Reis et al. [10] reported a collection from Santana de Pirapama, Brazil. This indicates research interest beyond the species’ natural range. Such findings also point to opportunities for conservation and cultivation in non-native regions [27].

Figure 3b presents the distribution of author affiliations linked to *O. indicum* publications. India, China, and Thailand dominate, but additional contributions come from the United States, Malaysia, and Japan. These affiliations demonstrate that while research is concentrated in the plant’s native range, international collaboration and global attention are gradually expanding.

#### 3.1.3. Top Journals

Overall, 110 journals have published studies on *O. indicum* (Table 1). The leading outlets are the Journal of Ethnopharmacology (18 articles), Molecules (11), and Natural Product Research (6). Most of the highly represented journals are pharmacology- or phytochemistry-oriented, which reflects the dominance of biomedical approaches. A smaller number of contributions appeared in food- and nutrition-focused outlets such as Food Chemistry, Nutrients, and the Journal of Functional Foods. Yet, during the full-text screening stage of our review, studies in these journals primarily reported phytochemistry or pharmacological assays rather than food applications. This distribution indicates that although *O. indicum* is gaining visibility across disciplines, its potential as a functional food ingredient remains underexplored.

#### 3.1.4. Top-Cited Documents

**Figure 4:**
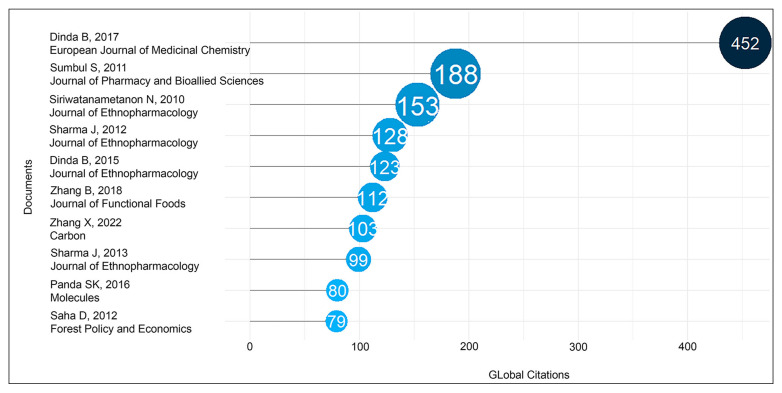
Most global cited documents related to *O. indicum* [8,20,28,29,30,31,32,33,34,35].

Figure 4 shows the top-cited papers related to *O. indicum*. The top article was by Dinda et al. [28] (452 citations), which examined the therapeutic potential of baicalin and baicalein against inflammatory disorders [28]. Two highly cited reviews, Sumbul et al. [29] (188 citations) and Dinda et al. [20] (123 citations), provided broad syntheses of phenolic compounds and therapeutic *O. indicum* potentials, respectively [20,29]. The prominence of these reviews shows that review articles play a key role in bringing together scattered findings and guiding new research directions. Yet, no review has mapped the traditional uses with scientific health effects. In addition, none of the most-cited works addressed sensory evaluation, formulation, or dietary application, underscoring that food-oriented research on *O. indicum* has not shaped the field to date.

#### 3.1.5. Author Keywords and Thematic Development

**Figure 5:**
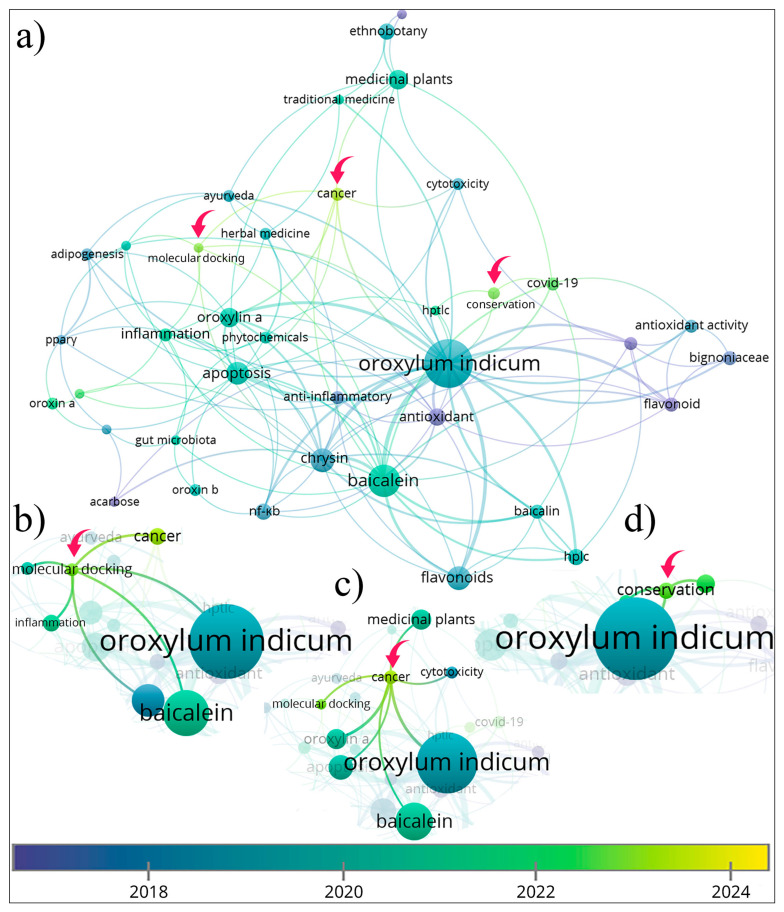
Thematic map related to studies on *O. indicum*. Panel (**a**) is the overall keyword co-occurrence network, where node color corresponds to the average publication year and node size indicates occurrence frequency. The arrows highlight new topics (yellow clusters). Panels (**b**–**d**) are zoomed-in views of cropped regions from panel (**a**) for clearer visualization of individual clusters.

Figure 5 is the result of a co-occurrence analysis of author keywords (a minimum threshold of 3), which yielded 39 terms. The two terms with the highest frequency are “*Oroxylum indicum*” (65 occurrences) and “baicalein” (29 occurrences). This data indicates the central role of this specific flavonoid in the current research domain.

The keyword “*Oroxylum indicum*” demonstrates extensive connectivity with other terms, including cancer, Ayurveda, COVID-19, baicalein, baicalin, and oroxylin A, which shows a strong emphasis on medicinal benefits. The second most frequent keyword is baicalein, which is clustered with *O. indicum*, COVID-19, cancer, inflammation, acarbose, and NF-κB (nuclear factor kappa-light-chain-enhancer of activated B cells). It is consistent with its reported anticancer, anti-inflammatory, and antidiabetic activities [22]. Figure 5b–d show the three keyword clusters in the most recent studies. The keyword “COVID-19” appeared the most during 2022 (average of publication years). However, since COVID-19 is no longer a concern, research on the herb for treating the virus has declined. Recent clusters highlight molecular docking, cancer, and conservation. It can be observed that researchers increasingly use molecular docking to study *O. indicum* compounds, especially baicalein, in cancer treatment. In addition, conservation of the plants is also of concern. Kumari et al. [36] reported the tree is endangered because of the destruction of its natural habitat.

**Figure 6:**
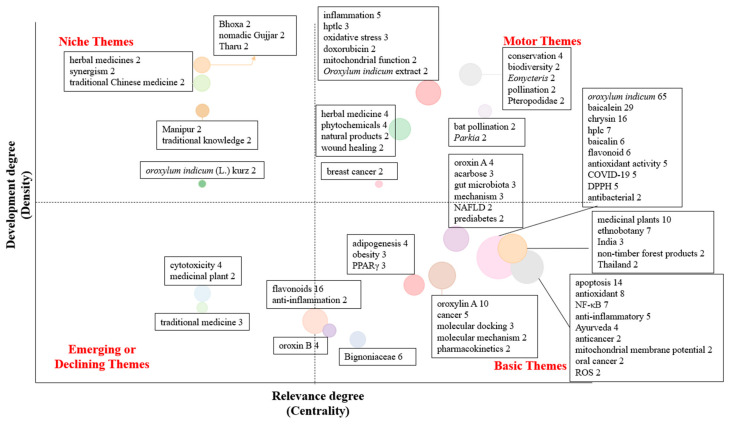
Thematic map related to studies on *O. indicum*. Bubble size represents the number of documents associated with each theme, while colors differentiate distinct thematic clusters.

The thematic map (Figure 6) further organizes research directions. Research topics are positioned based on their relevance (centrality) and level of development (density). Niche themes are specialized areas that are well developed but not strongly connected to other topics. Some main keywords are nomadic Gujjar (location name), Bhoxa (location name), Manipur (location name), Tharu (location name), traditional knowledge, herbal medicines, and traditional Chinese medicine. These suggest that studies have been well developed on localized ethnobotanical or ethnomedicinal knowledge. Motor themes are highly relevant and actively studied topics. Some main keywords are biodiversity, conservation, Pteropodidae (bat family), and pollination, which are closely related to ecological and environmental context. Emerging or declining themes have low development and low relevance, suggesting they are either new areas just starting to appear or older topics losing importance. Most importantly, basic themes are the foundations of research on *O. indicum*. They are widely explored, yet less detailed. Some main keywords are cancer, molecular docking, baicalein, baicalin, oroxylin A, and chrysin. Together, keyword and thematic analyses reveal a strong biomedical orientation, with growing attention to conservation, but little evidence of food applications, sensory evaluation, or dietary studies.

### 3.2. Research Classification Via the ADO Framework

**Figure 7:**
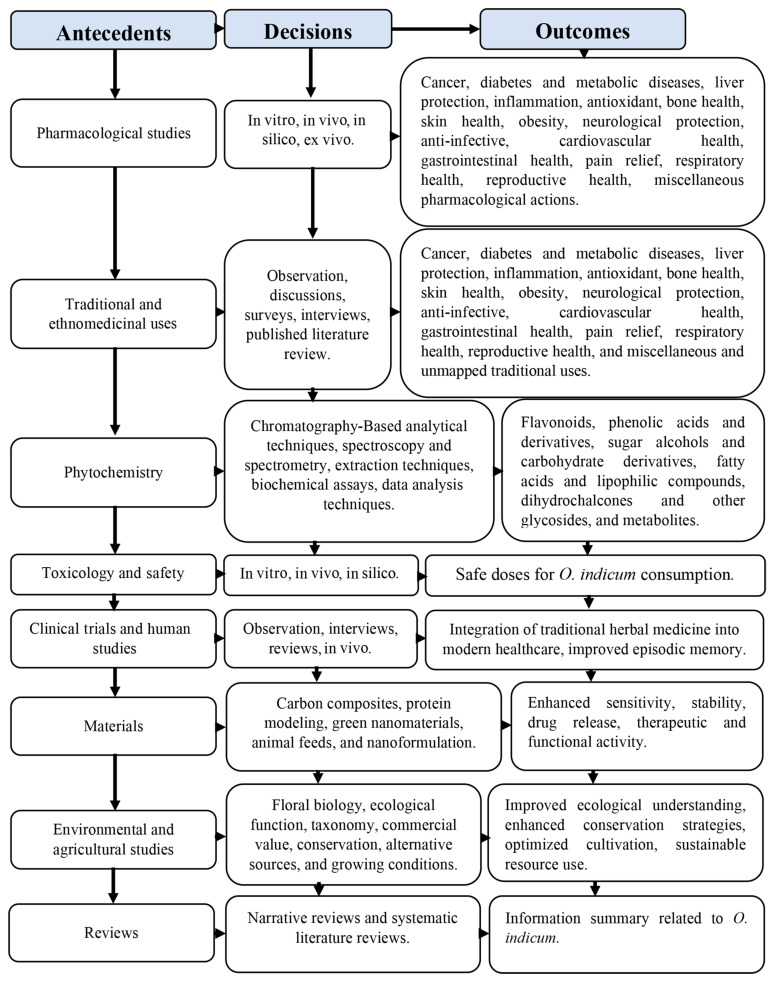
ADO framework for research related to *O. indicum*.

As noted in the top journals section, most studies on *O. indicum* focus on phytochemistry and pharmacology, with little attention to food applications. Besides descriptive patterns, we applied the Antecedents–Decisions–Outcomes (ADO) framework to classify research motives, methodological choices, and reported outcomes (Figure 7). This approach helps reveal not only what types of studies exist, but also how they were designed and what they achieved.

Manual screening identified eight main antecedent categories: (1) pharmacological studies, (2) traditional and ethnomedicinal uses, (3) phytochemistry, (4) toxicology and safety, (5) clinical trials and human studies, (6) materials, (7) environmental and agricultural studies, and (8) reviews. Three papers were grouped as “Others,” since *O. indicum* appeared only as part of multi-herb formulations or a reference in substitution without plant-specific methods or outcome relevance.

### 3.3. Pharmacological Studies, Ethnomedicinal Uses, and Mapping

A total of 106 studies were assigned to the pharmacological category (Table 2). These investigations tested numerous biological effects of *O. indicum* using extracts from different plant parts, including stem bark, pods, flowers, seeds, leaves, fruits, roots, and root bark. Ethanolic and methanolic extracts by decoction or maceration were the most common preparations, while some studies tested purified flavonoids. Consistent with the keyword analysis (Figure 5), the leading bioactive compounds were baicalein, baicalin, chrysin, oroxylin A, oroxin A, oroxin B, and their derivatives [1,9,37,38,39]. Above all, baicalein was frequently associated with liver protection, bone health, neuroprotection, pain relief, anti-obesity, antidiabetic, anti-infective, anti-inflammatory, antioxidant, and anticancer activities [8,14,40,41,42,43] (Table 2).

The methodologies used covered in vitro assays, in vivo models, in silico studies, and a limited number of ex vivo experiments. In vivo investigations provide the most biologically relevant evidence, as they reflect physiological processes such as immune modulation, metabolism, and absorption. Encouragingly, nearly every pharmacological category had at least one in vivo study with the exception of anti-infective and obesity-related research. The absence of in vivo antimicrobial research may be due to biosafety and ethical complexities in infection models. In contrast, the absence of in vivo obesity models is an indication of research gaps rather than methodological difficulties. This interpretation is supported by the thematic map (Figure 6), which places obesity in the “emerging” category. Given the significant global prevalence of obesity and the promising in vitro data obtained, the development of in vivo animal models and subsequent human clinical trials should be established as a research priority.

Table 3 shows the ethnomedicinal uses of *O. indicum*, many of which are well scientifically validated with pharmacological activities. The traditional uses are classified into the subgroups of pharmacological activity categories for easy comparison. The degree of alignment between ethnomedicinal applications and experimental data can be described across three levels: strongly supported, moderately supported, and unclear or unsupported.

At the highest level of alignment, there are cancer, diabetes, liver protection, anti-inflammatory activity, antioxidant effects, cardiovascular health, gastrointestinal health, pain relief, and respiratory health (Table 3). For example, Lalou et al. [37] reported that *O. indicum* had anti-tumor effects, which supports its traditional use in cancer treatment. Similarly, antidiabetic activity was validated by Hengpratom et al. [42], Mei et al. [94], and Mangal et al. [93], aligning with folk remedies for diabetes. Arthritis and rheumatism can be grouped under either bone health or anti-inflammatory. However, both categories are strongly supported by in vivo studies showing NF-κB (nuclear factor kappa-light-chain-enhancer of activated B cells), 5-LOX (arachidonate 5-lipoxygenase), and COX-2 (cyclooxygenase-2) inhibition [2,39] and anti-osteoporotic effects [12,14,90,91,92].

Traditional concepts such as “detoxification” or “rejuvenation” also map well to demonstrated antioxidant, cytoprotective, and membrane-stabilizing activities. These traditional terms are not clearly defined, yet their meanings generally match how the plant helps reduce oxidative stress and support cell health. Heart-related problems are supported by Yuvaraj et al. [97] and Pondugula et al. [18]. They found that *O. indicum* protects the heart, reduces artery buildup, lowers fat levels in the blood, and fights inflammation and harmful oxidation. Respiratory uses, particularly asthma, cough, and allergy, are directly supported [121]. Conditions like laryngitis or tonsillitis lack direct evidence. However, the herbs’ anti-inflammatory properties might be somewhat helpful in non-infectious or chronic cases. Hepatoprotective activity is also consistent with traditional uses for liver ailments [19], though direct validation for jaundice and hepatitis is lacking. Other groups, such as gastrointestinal disorders and pain relief, are similarly well supported, as summarized in Table 3. Taken together, these overlaps provide strong scientific confirmation for most of the plant’s major traditional applications.

In contrast, some traditional uses are moderately supported, including skin health, obesity, neurological protection, anti-infective, and reproductive health. Skin disorders such as leukoderma remain unsupported, whereas wound healing has some backing. Obesity was studied with in vitro and in silico research, but in vivo studies are needed. For neurological protection, no direct experimental models have assessed seizure activity, though neuroprotective studies provide partial support. Anti-infective claims are mixed. Its effectiveness against intestinal worms is strongly supported by lab studies. Pneumonia also has strong support, mainly due to its action against *Staphylococcus aureus*. Nevertheless, effects on fever, typhoid, and cholera are only indirectly supported. Antiviral studies against DENV-2 (Dengue Virus serotype 2) [112], ZIKV (Zika Virus) [10], and SARS-CoV-2 (Severe Acute Respiratory Syndrome Coronavirus 2) [15] partly match uses for measles and smallpox, but malaria and tuberculosis lack evidence.

Some uses remain unsupported or unmapped, including tonic, hair tonic, dropsy, urinary problems, scrotal swelling, dog bite, enlarged spleen, antipyretic, hemorrhage, and scorpion sting. These indicate the breadth of traditional knowledge but require new research to explore their biological basis. In addition, new activities not described traditionally have emerged, such as anti-gout [124] and anti-sickling [125], showing that modern pharmacology is expanding beyond recorded ethnomedicinal claims.

### 3.4. Phytochemistry

Phytochemical investigations of *O. indicum* focus on three objectives, including active compound identification, linking to biological effects, and extraction method improvement (Table S3). Techniques, such as chromatography (high-performance liquid chromatography (HPLC) and liquid chromatography-mass spectrometry (LC-MS)), spectroscopy (nuclear magnetic resonance (NMR)), and multivariate analysis, are commonly used [37,42,66]. In addition, newer eco-friendly approaches like Natural Deep Eutectic Solvent–Ultrasound-Assisted Extraction (NADES–UAE) are also used to enhance flavonoid yield and stability [84]. Throughout studies, flavonoids dominate the chemical profile. Baicalein, baicalin, chrysin, oroxylin A, oroxin A, oroxin B, and their derivatives are the most consistently reported. Some less frequently appearing flavonoids are apigenin, scutellarein, tetuin, luteolin, hispidulin, and their derivatives. These compounds have been linked to different health effects, such as liver protection, skin health, bone health, neurological protection, antioxidant, anti-inflammatory, antidiabetic, and anticancer effects, which explains their central role in the pharmacological activities of the plant.

Some mechanistic studies have discovered how *O. indicum* flavonoids exert their bioactivities. The antidiabetic effects of baicalein are by modulating gut microbiota and improving metabolic function in diabetic rodent models [8]. Baicalin shows Src kinase (Src tyrosine kinase) and reduces IL-6 (interleukin-6) production, indicating anti-inflammatory effects at the molecular level [89]. Chrysin activates the p53 pathway to induce apoptosis in cancer cells with no genotoxic stress [7]. Oroxylin A gives anti-adipogenic and lipolytic activity Via PPARγ (peroxisome proliferator-activated receptor gamma) suppression [95]. Meanwhile, oroxin A improves porcine embryo development by enhancing blastocyst formation and mitochondrial function while reducing ROS (reactive oxygen species), apoptosis, and autophagy [123]. In contrast, oroxin B has anti-osteoclastogenic and anti-osteoporotic activity in ovariectomized mice by the regulation of the MAPK/NF-κB (mitogen-activated protein kinase/nuclear factor kappa) pathway [91].

**Figure 8:**
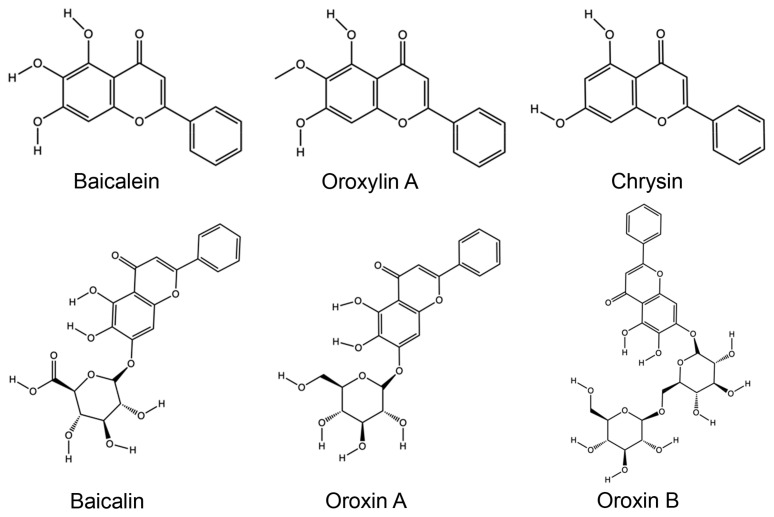
Chemical structures of the major flavonoids in *O. indicum* (baicalein, baicalin, chrysin, oroxylin A, oroxin A, and oroxin B).

In general, the phytochemical profile of *O. indicum* indicates strong potential for developing functional food ingredients, provided challenges of bitterness and bioavailability can be addressed. Figure 8 shows the chemical structures of the major flavonoids in *O. indicum*, including baicalein, baicalin, chrysin, oroxylin A, oroxin A, and oroxin B.

### 3.5. Toxicology, Safety, Clinical Trials, and Human Studies

In general, preclinical studies show that *O. indicum* is safe at the tested doses (Table S4). Acute, subacute, and in vivo assessments have reported no major adverse effects or organ damage. For example, Singh and Kakkar [70] observed no toxicity in diabetic or normal rats following a 28-day oral administration of stem bark extract at 250 mg/kg/day. Similarly, a single oral dose of 2,000 mg/kg of methanolic bark extract produced no mortality or visible toxicity in Wistar albino rats over two weeks [118]. These findings are consistent with its long-standing traditional use and indicate a broad safety margin. However, most studies were restricted by short-term exposure (≤28 days). There is little information on chronic, reproductive, or developmental toxicity. Addressing these gaps is essential for supporting wider applications.

Human evidence is very limited. Chotchoungchatchai et al. [133] conducted an ethnopharmacological interview and survey documenting its use in Thai traditional medicine for cough, fever, and fatigue. The research indirectly supports its safety profile without clinical endpoints. The only controlled human trial to date was conducted by Lopresti et al. [100]. A standardized extract (Sabroxy^®^, Sabinsa Corporation, East Windsor, NJ, USA) was used in a placebo-controlled study with people who self-reported having cognitive problems. Participants reported improved mood, thinking, and memory after 12 weeks (doses of 1000 mg daily), with no significant adverse effects. Together, these studies link lab research to human use. Although such findings are encouraging, they remain preliminary. Larger, more well-controlled clinical trials are needed to confirm how well it works, the right dose, and long-term safety.

Within the context of functional food, available data suggests that *O. indicum* is not acutely toxic. However, its safety at typical dietary consumption levels has not been confirmed. Most pharmacological investigations use extracts or purified compounds at pharmacological dosages and they are far higher than what might be obtained by food intake. To bridge this research gap, the implementation of standardized toxicity protocols, dose–response research at dietary levels, and human clinical validation are required to verify its suitability for functional food applications.

### 3.6. Food Applications

#### 3.6.1. Traditional and Current Food Uses

*O. indicum* has been consumed as food in Southeast Asia for a long time. In Malaysia, its young leaves and fruits are sold in local markets and eaten raw as salad (ulam) [22]. In Thailand, Laos, and Northeastern India, immature fruits and shoots are commonly eaten as vegetables [111]. They are often roasted or served with other dishes [9]. Despite this traditional usage, the plant’s nutritional composition, cooking safety, and culinary potential remain poorly described. Only one study by Choonong et al. [9] examined how conventional grilling affected flavonoid concentrations, but this did not address food product development. Meanwhile, severe astringency and bitterness remain the major barriers to wider consumption [135]. Many studies suggest that flavonoid-rich foods have intense bitter tastes and could affect consumer acceptance [136].

#### 3.6.2. Bitterness as a Critical Challenge

Bitterness is one of the least understood basic tastes and can arise from a variety of compounds, including amino acids, peptides, esters, lactones, phenols, polyphenols, flavonoids, terpenes, and alkaloids [135]. In fruits and vegetables, phenolic compounds, especially flavonoids, are commonly the main contributors [135]. Behrens et al. [137] showed that baicalein and baicalin activate human bitter taste receptors TAS2R14. Meanwhile, chrysin is present in other bitter foods such as apricot seeds [138] and bitter melon [139]. This suggests a possible link between the health-promoting flavonoids of *O. indicum* and its bitterness. If flavonoids are the major drivers, reducing bitterness without losing nutritional and pharmacological value will be difficult.

#### 3.6.3. Approaches to Detect and Manage Bitterness

Bitter compounds can be studied using both sensory and instrumental methods. Analytical techniques, such as HPLC or LC–MS, can be correlated with human sensory taste intensities or electronic tongue, which will indicate the main bitterness contributors. Meanwhile, computational approaches like molecular docking can predict interactions between flavonoids and bitter taste receptors [140]. In general, three methods are used to reduce bitterness, including physicochemical interactions, receptor-level inhibition using bitter blockers, and cognitive mixture suppression [135]. However, processing methods like high temperature or alkalization may reduce bitterness but also degrade bioactive compounds [135]. The traditional Thai roasting method to prepare *O. indicum* could reduce the bitterness, yet it may also deteriorate its heat-sensitive flavonoids. More promising strategies involve bitterness masking, which improves food flavor and retains bioactive bitter compounds. Sharafi et al. [141] showed that sodium acetate, sodium chloride, and aspartame could partially mask bitterness in vegetables. However, masking vegetable bitterness depends on vegetable type and taste phenotype. Ke et al. [142] used sweeteners and amino acids to suppress bitterness in *Zanthoxylum bungeanum*, but it was only effective to a certain extent. More recently, people have turned to computer-based techniques such as molecular docking for understanding compound-receptor mechanisms and pharmacophore for discovering inhibitors. Kan et al. [143] found that egg protein-derived peptides can bind TAS2R14 receptors, effectively reducing perceived bitterness. The incorporation of taste-masking agents with receptor inhibitors may therefore be a thorough approach to both reduce bitterness and retain nutritional values of *O. indicum* products.

#### 3.6.4. Consumer Perception and Acceptability

Not all bitter foods are rejected. Coffee, dark chocolate, and red wine are widely consumed with their bitter taste [135]. Acceptance depends on genetic differences in bitter taste perception (PTC/PROP non-tasters, medium tasters, and supertasters) as well as cultural exposure and repeated dietary contact [135]. In addition, access to health-related information is another significant factor that can increase consumer acceptance [144], particularly within an Asian cultural context where a common belief holds that the more bitter the food, the healthier it is. This suggests that consumer testing is important to determine acceptable levels of *O. indicum* bitterness in functional food prototypes. Sensory techniques, such as threshold analysis, hedonic scaling, and just-about-right (JAR) scaling, can be taken to identify the balance between bitterness reduction and health perception. Such approaches will help determine whether bitterness should be suppressed, tolerated, or even leveraged as part of a “healthy-bitter” product identity.

#### 3.6.5. Future Directions for Food Applications

The phytochemical, pharmacological, and ethnomedicinal evidence indicates strong potential for *O. indicum* as a functional food ingredient. However, most efficacy studies were done in vitro or in animal models at pharmacological doses, and only two human studies exist. For functional food development, safety at dietary intake levels must first be established. Then, discovering the compounds responsible for bitterness will guide targeted masking or formulation strategies. Finally, consumer studies should be carried out to determine acceptable bitterness thresholds and preferred product formats, such as snacks, beverages, or extracts. Integrating safety validation, standardization, bitterness management, and consumer insights can support the use of *O. indicum* in a functional food resource.

### 3.7. Material, Environmental, and Agricultural Studies

In materials science, the plants have been applied in carbon composites, protein modeling, green nanomaterials, animal feeds, and nanoformulation. Mim et al. [80] used plant extracts to make cerium oxide nanoparticles. Worakitjaroenphon et al. [145] made silver and gold nanoparticles using a microwave-assisted green synthesis method. Beyond nanoparticle synthesis, *O. indicum* has also been incorporated into biomass-derived carbon materials. For example, Zhang et al. [34] used its biomass to create a 3D micro-nanostructure. This material showed good conductivity and structural properties. Greene et al. [146] studied the use of *O. indicum* as medicinal feed for elephants.

In the environmental and agricultural domain, research has explored the plant’s role in floral biology, ecological function, taxonomy, commercial value, conservation, alternative resource potential, and growing conditions. Sritongchuay et al. [147] found that *O. indicum* only reopens after receiving pollen from another plant, not from the same one. Sonia et al. [148] employed leaf anatomy to categorize *O. indicum* and other Bignoniaceae plants. Saha et al. [35] discovered that *O. indicum* is a significant non-timber forest product in northeast India. It benefits rural income and indigenous populations. Kumari et al. [36] stressed the critical necessity to protect medicinal plants such as *O. indicum* because increasing habitat degradation, overexploitation, and lack of systematic cultivation are pushing many valuable species toward vulnerability or extinction.

## 4. Conclusions

*O. indicum* is a promising yet underexploited resource for functional food development. Its stem bark, pods, flowers, seeds, leaves, fruits, roots, and root bark are already consumed in many parts of Southeast Asia. This is the first systematic review that integrated the PRISMA framework, bibliometric mapping, and ADO framework for a comprehensive overview of *O. indicum*. The PRISMA framework was first used for relevant study selection, while bibliometric analysis indicated overall trends. The ADO framework helped to further classify selected studies based on their objectives, methods, and outcomes. It also indicated health effects supported partly or fully by in vitro/in silico/in vivo research. The findings highlight that flavonoids such as baicalein, baicalin, chrysin, oroxylin A, oroxin A, oroxin B, and their derivatives are frequently recognized as the dominant bioactive compounds. These compounds are linked to many validated pharmacological effects, including liver protection and anticancer activities. Mapping traditional knowledge with experimental data shows that many folk uses of the plant, such as for treating cancer, diabetes, liver ailments, and respiratory conditions, are strongly supported by science. However, other uses are only moderately supported or have not been scientifically researched yet.

Despite its rich bioactive profile and cultural history as a food, research on *O. indicum* is still dominated by phytochemistry and pharmacological assays. Although this review summarizes extensive evidence, most studies differ considerably in sample size, methodological rigor, and dosing strategies. Only a few investigations have used in vivo models, and human clinical trials are still lacking. These limitations highlight the need for more standardized and clinically relevant research designs. Furthermore, nearly all efficacy studies rely on pharmacological doses rather than dietary intake. Bitterness and astringency are also major obstacles for food applications, with little research on sensory evaluation, consumer acceptance, or formulation strategies.

Future work should focus on health benefits that are not yet strongly supported by science, such as conducting in vivo studies for obesity. Since short-term toxicity has been evaluated, studies on long-term safety, reproductive outcomes, and human clinical trials should be considered (1). Standardized toxicity protocols at dietary levels, particularly with human subjects, should be next (2). Determining whether beneficial flavonoids like baicalein and baicalin are major bitterness contributors is essential for developing bitterness removal strategies (3). Following that, testing effective masking and receptor-inhibition strategies will be needed (4). Finally, consumer-focused studies will help establish acceptable sensory thresholds and product formats (5). Integrating these approaches will make the safe and effective transition of *O. indicum* from a traditional herb into a scientifically validated functional food ingredient.

## Figures and Tables

**Table 1:** Top 35 journals publishing the selected articles on *O. indicum* (number of publications in parentheses).

Top 35 Journals and Number of Publications		
Journal of Ethnopharmacology (18)	Cancers (2)	Journal of Chemistry (2)
Molecules (11)	Current Science (2)	Journal of Functional Foods (2)
Natural Product Research (6)	Economic Botany (2)	Journal of Natural Medicines (2)
Journal of Ayurveda and Integrative Medicine (5)	Ethnobotany Research and Applications (2)	Journal of Pharmaceutical and Biomedical Analysis (2)
Phytomedicine (4)	European Journal of Pharmacology (2)	Journal of Pharmacy and Bioallied Sciences (2)
European Journal of Medicinal Chemistry (3)	Fitoterapia (2)	Medicinal Chemistry Research (2)
Heliyon (3)	Food Bioscience (2)	Pharmacological Research-Modern Chinese Medicine (2)
Indian Journal of Traditional Knowledge (3)	Food Chemistry (2)	Plants (2)
Journal of Biomolecular Structure and Dynamics (3)	Frontiers in Pharmacology (2)	PLoS ONE (2)
Nutrients (3)	Industrial Crops and Products (2)	Processes (2)
Research Journal of Pharmacy and Technology (3)	Journal of Applied Pharmaceutical Science (2)	RSC Advances (2)
Biomedicine and Pharmacotherapy (2)	Journal of Applied Research on Medicinal and Aromatic Plants (2)	

**Table 2:** Summary of representative pharmacological activities of *O. indicum* groups.

Group	Related Activities	Extract/ Compound	Plant Part	Model	References
Cancer	anticancer, anti-tumor, cytotoxic, anti-metastatic, histone deacetylase (HDAC) inhibition, and anti-lymphoma.	Endophytic fungus extract, ethanol extract, ethyl acetate extract, methanol extract, aqueous extract, hydroalcoholic extract, fungal endophyte metabolites, baicalein, chrysin, oroxylin A, oroxylin A glycoside, oroxylin A-7-O-glucuronide, oroxyquinone, methoxy-chrysin, scutellarein-7-rutinoside, oroxin B, chrysin-7-O-glucuronide, oroxindin, oroxin A, and semi-synthetic derivatives.	Stem bark, bark, leaves, fruit, young stems, root, and root bark.	In vitro, in vivo, in silico.	[7,13,30,37,40,44,45,46,47,48,49,50,51,52,53,54,55,56,57,58,59,60,61,62,63]
Diabetes	Anti-prediabetes, antidiabetic, and anti-NASH (non-alcoholic steatohepatitis).	Methanol extract, seed extract, baicalein, chrysin, baicalein-7-O-glucoside, baicalein-7-O-diglucoside, baicalin, oroxylin A, flavone derivatives, and glycosides.	Seed, stem bark, root, and bark.	In vitro, in vivo, in silico.	[8,38,64,65,66,67,68,69,70,71]
Liver Protection	Hepatoprotective and MAFLD (metabolic associated fatty liver disease).	Ethanol extract, apigenin, baicalein, chrysin, oroxylin A, scutellarin, tetuin, oroxin B, and oroxin A.	Stem bark and seed.	In vitro, in vivo, in silico.	[19,41,72]
Skin Health	Wound healing and skin-whitening.	Ethanol extract, aqueous extracts, and chrysin.	Leaf, seed, and stem bark.	In vitro, in vivo.	[73,74,75]
Antioxidant	Antioxidant.	Methanol extract, aqueous extract, ethanol extract, acetone extract, hexane extract, SCeCO_2_eH extract (supercritical carbon dioxide integrated hydrothermal extract), hydrothermal extract, Soxhlet methanol extract, cerium oxide nanoparticles, baicalin, chrysin, baicalein, oroxylin A, scutellarein, baicalein-7-O-gentiobioside, baicalein-7-O-glucoside, and hispidulin.	Bark, fruit, seed, leaf, branch, stem, twig, root, and immature pod.	In vitro, in vivo, in silico.	[11,76,77,78,79,80,81,82,83,84,85,86]
Anti-inflammatory	Anti-inflammatory.	Ethanol extract, dichloromethane extract, ethyl acetate extract, decoction, baicalein, scutellarein, oroxylin A, chrysin, hispidulin, oroxin A, and baicalin.	Bark, leaves, seeds, shoots, fruits, grilled fruits, stem bark, and root bark.	In vitro, in vivo, in silico.	[2,9,16,17,39,87,88,89]
Bone Health	Anti-osteoarthritic, anti-osteoporotic, osteogenic support, and skeletal protective activity.	Ethanol extract, oroxin B, oroxylin A, baicalein, and other major flavonoids.	Stem bark.	In vitro, in vivo, in silico.	[12,14,90,91,92]
Obesity	Anti-adipogenic and anti-obesity.	Ethyl acetate extract, ethanol extract, baicalein, oroxylin A, luteolin, apigenin, and chrysin.	Fruit and bark.	In vitro, in silico.	[42,93,94,95]
Cardiovascular Health	Anti-atherosclerotic and cardioprotective.	Sabroxy (*O. indicum* extract), methanol extract, and chrysin.	Stem bark and root bark.	In vivo.	[18,96,97]
Gastrointestinal Health	Anti-ulcer.	Dihydrooroxylin A-7-O-methyl glucuronide and chrysin.	Stem bark.	In vivo.	[98]
Neurological Protection	Neuroprotective, neuroregenerative, anti-Alzheimer, antidepressants, and anti-neuroinflammatory	Sabroxy, water extract, methanol extract, ethanol extract, baicalein, oroxylin A, chrysin, hispidulin, apigenin, baicalin, and isoverbascoside.	Leaf, bark, seed, root, root bark, and fruit pod.	In vitro, in vivo, in silico.	[43,99,100,101,102,103,104,105,106,107,108]
Anti-infective	Antibacterial, antimicrobial, antiviral, anthelmintic, and antifungal.	Aqueous extract, methanol extract, ethanol extract, *Lactobacillus plantarum* WU-P19, baicalein, baicalin, 5,6,7-trimethoxyflavone-8-O-β-D-glucopyranoside, oroxylin A-7-O-β-D-glucuronide butyl ester, chrysin, 6-methoxybaicalein, oroxylin A-7-O-glucoside, and oroxylin A.	Seeds, young fruits, flowers, fermented fruit, leaf, stem, bark, and root.	In vitro, in silico.	[10,15,31,109,110,111,112,113,114,115,116,117,118]
Pain Relief	Anti-nociceptive.	Ethanol extract, water extract, and baicalein.	Stem barks and roots.	In vitro, in vivo, in silico.	[119,120]
Respiratory Health	Anti-allergic and anti-asthmatic.	Oroxylin A.	Not specified.	In vitro, in vivo.	[121]
Reproductive Health	Antifertility and embryo support	Aqueous extract, methanol extract, and oroxin A.	Stem bark.	Ex vivo, in vivo, in vitro.	[122,123]
Miscellaneous Pharmacological Actions	Pharmacokinetics, anti-gout, and anti-sickling	Ethanol extract, methanol extract, baicalin, oroxylumoside A, oroxylumoside B, darendoside A, and leucosceptoside A.	Seeds and stem bark.	In vitro, in vivo, ex vivo	[124,125,126]

Due to many studies having an extensive number of pharmacological activities tested, only one representative activity in each study is listed here and is used for grouping. A full dataset of all screened studies is provided in Table S1.

**Table 3:** Summary of ethnomedicinal uses of *O. indicum*.

Group	Ethnomedicinal Uses	Comparison with Pharmacological Activities	References
Cancer	Cancer.	Strongly supported	[3]
Diabetes	Antidiabetic.	Strongly supported	[3,93,127,128]
Liver Protection	Jaundice, liver problems, hepatitis, and hepatoprotective.	Strongly supported	[3,4,32]
Skin Health	Leukoderma, urticaria, infantile erythema, cuts and wounds, burns, skin disorder, and skin diseases.	Moderately supported	[3,5,129]
Anti-inflammatory	Arthritis, inflammation, and rheumatism.	Strongly supported	[3,4]
Antioxidant	Detoxification and rejuvenation.	Strongly supported	[2,93]
Bone Health	Arthritis and rheumatism.	Strongly supported	[3,4]
Obesity	Obesity.	Moderately supported	[93]
Cardiovascular Health	Cardiac disorders, high blood pressure, hypertension, and heart problems.	Strongly supported	[3,129,130]
Neurological Protection	Headache, neuralgia, epilepsy, and paralysis.	Moderately supported	[3,33,131]
Gastrointestinal Health	Stomach problems, diarrhea, carminative, stomachache, dysentery, purgative, astringent, stomachic, dyspepsia, gastropathy, gastralgia, stomach cleaning, colic, indigestion, bloody stool, stomach ulcer, constipation, abdominal pain, gastric ulcer, ulcer, piles, colitis, hemorrhoids, and other digestive ailments.	Strongly supported	[3,4,5,6,131,132]
Anti-infective	Fever, malaria, tuberculosis, smallpox, cholera, measles, typhoid, gonorrhea, pneumonia, and anthelmintic.	Moderately supported	[3,5,129]
Pain Relief	Muscle pain, chest pain, body pain, sprains, and analgesic.	Strongly supported	[3,4,120]
Respiratory Health	Cough, bronchitis, pharyngodymia, asthma, sore throat, laryngitis, hoarseness, allergic disease, and tonsillitis.	Strongly supported	[3]
Reproductive Health	Placental problem, menstrual disorders, womb ailment, leucorrhea, and a blood tonic.	Moderately supported	[3,4,129,133]
Miscellaneous and Unmapped Traditional Uses	Hair tonic, tonic, dropsy, urinary problems, scrotal swelling, and dog bite, enlarged spleen, antipyretic agent, hemorrhage, and scorpion sting	Unclear or unsupported	[3,4,5,120,134]

A full dataset of all screened studies is provided in Table S2. “Strongly supported” indicates direct and consistent validation through experimental studies, especially in vivo. “Moderately supported” refers to partial or indirect evidence (e.g., in vitro or in silico only, or limited relevance). “Unsupported” includes uses with no scientific evidence or too vague to be pharmacologically assessed.

## Data Availability

The original contributions presented in this study are included in the article/Supplementary Materials. Further inquiries can be directed to the corresponding author.

## Supplementary Materials

The following supporting information can be downloaded at https://www.mdpi.com/article/10.3390/foods14203465/s1, Table S1: Studies on pharmacological activities of *O. indicum*; Table S2: Ethnomedicinal uses of *O. indicum*; Table S3: Studies on *O. indicum* phytochemistry; Table S4: Studies on *O. indicum* safety and toxicology. Additional references cited in the Supplementary Materials are included in the main reference list as [149,150,151,152,153,154,155,156,157,158,159,160,161,162,163,164,165,166,167,168,169].

## Abbreviations

The following abbreviations are used in this manuscript:

ADO	Antecedents–Decisions–Outcomes
PRISMA	Preferred Reporting Items for Systematic Reviews and Meta-Analyses
PPARγ	Peroxisome Proliferator-Activated Receptor Gamma
NAFLD	Nonalcoholic fatty liver disease
DPPH	2,2-diphenyl-1-picrylhydrazyl
HPTLC	High-Performance Thin-Layer Chromatography
ROS	Reactive Oxygen Species
